# ACHES AND PAINS: HOW DO THEY AFFECT TRANSITIONS FROM DRIVING?

**DOI:** 10.1093/geroni/igac059.2283

**Published:** 2022-12-20

**Authors:** Anne Barrett, Cherish Michael

**Affiliations:** Florida State University, Tallahassee, Florida, United States; Florida State University, Tallahassee, Florida, United States

## Abstract

Chronic pain, which affects more than 1 in 4 middle-aged and older adults, can have profound implications for everyday activities, like driving. Although research has revealed pain’s effect on driving performance, less is known about driving-related behaviors and self-assessments that are part of the process of transitioning from driving. We address this issue using data from an online survey of 3,441 Floridians aged 50 and older that was conducted between December 2020 and March 2021 and funded by the Florida Department of Transportation. We examine the association between pain and four driving-related outcomes: self-rated driving ability, self-regulated driving, perceived nearness of driving retirement, and planning for driving retirement. Results of multivariate regression analyses indicate that experiencing greater pain is associated with worse self-rated driving ability, more frequent self-regulated driving, and greater perceived nearness of driving retirement. Pain is not, however, associated with greater planning for driving retirement. These findings indicate that although greater pain may hasten the transition from driving, it may not lead to more planning for it. Both patterns suggest that pain may increase people’s risk of experiencing the social isolation that can follow driving retirement. By focusing on transitioning from driving, our study reveals a largely overlooked benefit of reducing pain – It could extend people’s years behind the wheel.

